# MaxEnt Modeling for Predicting the Potential Wintering Distribution of Eurasian Spoonbill (*Platalea leucorodia leucorodia*) under Climate Change in China

**DOI:** 10.3390/ani13050856

**Published:** 2023-02-26

**Authors:** Aihua Fu, Erhu Gao, Xiaoping Tang, Zengli Liu, Faxiang Hu, Zhenjie Zhan, Jiadong Wang, Xiaofeng Luan

**Affiliations:** 1 School of Ecology and Nature Conservation, Beijing Forestry University, No. 35 Tsinghua East Road, Haidian District, Beijing 100083, China; 2 Academy of Inventory and Planning, National Forestry and Grassland Administration, Beijing 100714, China

**Keywords:** waterbird, maximum entropy, wintering habitat, species’ range shift

## Abstract

**Simple Summary:**

Climate change has become an important cause of habitat loss. The Eurasian Spoonbill (*Platalea leucorodia leucorodia*) is a national grade II protected wildlife species in China. Few studies on the distribution and protection of Eurasian Spoonbill have been carried out in wintering grounds. We simulated the potential distribution of the Eurasian Spoonbill and analyzed the impact of climate change on this species, with occurrence sites recorded during the National Waterbird Simultaneous Survey of China in 2016 to support species conservation. We also analyzed the main environmental factors affecting its overwintering distribution. We conclude that the majority of suitable wintering habitats are concentrated in the middle and lower reaches of the Yangtze River and tend to shift northward under future emission scenarios. Moreover, the main factors affecting the suitable wintering habitat areas at present are water distance, altitude, precipitation, and mean temperature of the driest quarter. Therefore, future conservation work should pay considerable attention to the suitable habitat of the white spoonbill in the middle and high levels to effectively conserve this species over time. These findings offer a valuable reference for the conservation of the Eurasian Spoonbill and other endangered species under climate change. Furthermore, it provides a reference for wetland development, utilization, and protection.

**Abstract:**

Global climate change has become a trend and is one of the main factors affecting biodiversity patterns and species distributions. Many wild animals adapt to the changing living environment caused by climate change by changing their habitats. Birds are highly sensitive to climate change. Understanding the suitable wintering habitat of the Eurasian Spoonbill (*Platalea leucorodia leucorodia*) and its response to future climatic change is essential for its protection. In China, it was listed as national grade II key protected wild animal in the adjusted State List of key protected wild animals in 2021, in Near Threatened status. Few studies on the distribution of the wintering Eurasian Spoonbill have been carried out in China. In this study, we simulated the suitable habitat under the current period and modeled the distribution dynamics of the wintering Eurasian Spoonbill in response to climate change under different periods by using the MaxEnt model. Our results showed that the current suitable wintering habitats for the Eurasian Spoonbill are mainly concentrated in the middle and lower reaches of the Yangtze River. Distance from the water, precipitation of the driest quarter, altitude, and mean temperature of the driest quarter contributed the most to the distribution model for the wintering Eurasian Spoonbill, with a cumulative contribution of 85%. Future modeling showed that the suitable distribution of the wintering Eurasian Spoonbill extends to the north as a whole, and the suitable area shows an increasing trend. Our simulation results are helpful in understanding the distribution of the wintering Eurasian Spoonbill under different periods in China and support species conservation.

## 1. Introduction

The Intergovernmental Panel on Climate Change (IPCC) report showed that global warming temperature may be 1.5 °C higher than the preindustrial level by 2040. Moreover, it is projected to increase by nearly 0.2 °C every ten years [1]. Climate change is considered one of the crucial factors affecting biodiversity patterns and species distribution; it is also a major issue for conservationists [2,3]. Thus far, the distribution and population of species have significantly changed, even related to species extinction [3]. Previous studies have shown that as the climate warms, some species change their current suitable habitats and mainly migrate to high altitudes and high latitudes to adapt to the changing environment, whereas others lose their existing suitable habitats [4,5,6,7]. As sensitive species, birds are particularly affected by climate change because they are highly sensitive to climate and weather [8,9]. Shifts in bird distribution have already been linked to climate change [10,11,12,13]. Moreover, some studies have suggested that global bird and mammal populations have decreased considerably in regions with high temperatures [11]. Besides knowledge of the natural history and biology of a species, an understanding of the suitable habitat of a species is essential for species conservation and conservation planning [14,15]. Comparing the current and future predicted distributions of species enables conservationists to develop protection and project plans [2,16,17,18]. Therefore, scientifically understanding the impact of climate change on species distribution is crucial to adequately protect species in the context of climate change [19,20].

Species distribution models (SDMs), also known as ecological niche models, are essential tools in basic ecology and biogeography and are widely used in analyzing the impacts of climate change on species [14,21,22]. SDMs are one method to correlate species distribution information and relevant environmental variables and obtain the potential distribution of species through multiple algorithms based on niche theory [23,24]. The development of SDMs originated from the BIOCLIM model [25]. Then, many niche models, such as the generalized linear model (GLM), genetic algorithm for rule set production (GARP), random forest (RF), and maximum entropy (MaxEnt) were developed to predict species distributions. MaxEnt is widely used because it only needs species occurrence data to make high stability and high precision predictions under incomplete data and small sample size conditions [26,27,28,29]. It is also used to predict suitable habitats for birds [14,30,31], biodiversity loss [32], species conservation, and options for nature-protected areas [33,34].

The Eurasian Spoonbill is a common species and belongs to the Least Concern category under the IUCN Red List [35]. In China, it was listed as national grade II key protected wild animal in the adjusted State List of key protected wild animals in 2021, in Near Threatened status [36,37]. There are three subspecies, namely: *Platalea leucorodia leucorodia*, *Platalea leucorodia archeri*, and *Platalea leucorodia balsaci*. Among them, *Platalea leucorodia leucorodia* is distributed in China. The global population of the Eurasian Spoonbill (*Platalea leucorodia*) is approximately 63,000–65,000 [35]. Most countries have only a few hundred breeding pairs with a decreasing trend [38]. The Eurasian Spoonbill likes to live near wetlands, such as the banks of rivers, lakes, and reservoirs in open plains and mountainous and hilly areas, swamp wetlands, and offshore and coastal areas. It mainly winters in the lower reaches of the Yangtze River, South China, and southeast coastal areas [39,40]. As a migratory species, the Eurasian Spoonbill is more vulnerable to a series of specific threats (such as habitat loss/fragmentation, overexploitation, and climate change) than nonmigratory species, thereby hindering the sustainable existence of its population [35,41,42]. Climate change is one of the main factors determining bird distribution and geographic range change [8]. Studies on the European and African Eurasian Spoonbill showed that climate change alters the wintering migration distance, the distribution of suitable habitats, and impacts the protection network [43]. Most of the previous studies regarding the Eurasian Spoonbill have focused on breeding behavior, geographical distribution, living habitats and habitat selection, overwintering ecology, and distribution in local areas [44,45,46,47,48,49]. However, studies on the distribution of wintering habitats and the response and adaptation to climate change of the Eurasian Spoonbill in China are limited. Thus, relevant research is necessary to develop the protection work appropriately.

In this study, we used the MaxEnt model to simulate the potential suitable wintering habitat of the Eurasian Spoonbill. We also predicted the future influences on suitable wintering habitats under climate change scenarios for the 2050s and 2070s. In particular, we aimed to address the following questions: (1) Where is the current potential wintering distribution of the Eurasian Spoonbill? (2) What environmental factors affect the wintering distribution? (3) What is the impact of climate change on the current suitable wintering habitats? Our study will provide guidance for future field surveys and make conservation workers aware of the potential threat of climate change to this important species and other endangered species, thereby allowing suitable conservation and management work.

## 2. Materials and Methods

### 2.1. Source of Species Occurrence Data

**Figure 1:**
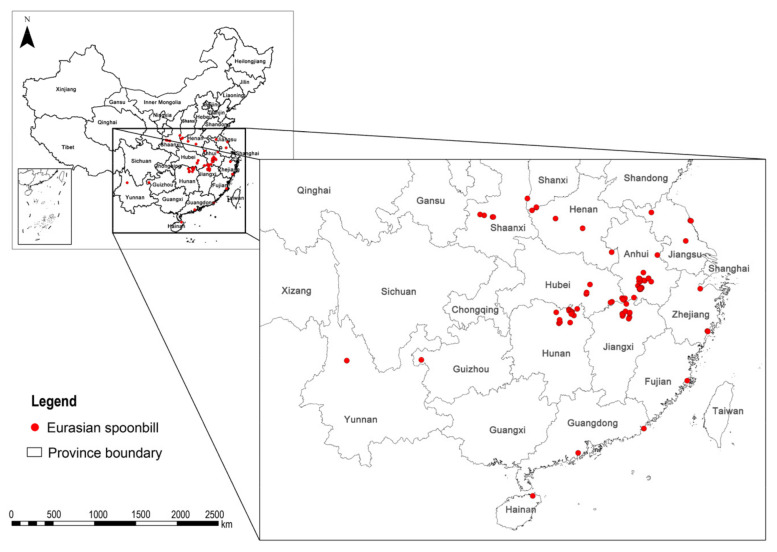
Eurasian Spoonbill occurrence sites in the National Waterbird Simultaneous Survey of China in 2016.

The Eurasian Spoonbill occurrence data used in this study were derived from the record during the Winter National Waterbird Simultaneous Survey of China in 2016 [50]. In this survey, 846 survey areas and 5255 observation points were determined based on the collection of historical data and literature, combined with the investigation and research on the migration and overwintering of migratory birds across the country, from 8 to 17 January 2016. The investigators conducted the survey synchronously using the unlimited distance point count method at the survey point with monocular (binocular) telescopes. The observation range is the longest distance that can be distinguished by a monocular telescope. Meanwhile, GPS devices were used to record the species’ locations. The synchronous survey here means that investigators used the same technical methods to conduct the investigation within 4 to 10 days [51]. A total of 105 sites of the Eurasian Spoonbills were found, involving 55 counties in 15 provinces. We selected only sites located at least 2 km from each to avoid spatial autocorrelation caused by clustered occurrences [52,53]. Finally, 87 locations for Eurasian Spoonbill occurrence points in China were obtained (Figure 1).

### 2.2. Environmental Variables

According to previous research reports, vegetation type, topography (altitude), climate, water distance, land-use type, and two human interferences (distance to road and villages) may affect the choice of wintering habitats for the Eurasian Spoonbill [54,55,56,57]. Therefore, we selected six categories of environmental datasets with 25 environmental variables, including 19 bioclimatic variables, 1 topographic variable (altitude), vegetation type variable, water distance variable, land-use type variable, and 2 human interference variables (distance to road and villages). Among these environmental variables, 19 bioclimatic layers were acquired from the WorldClim dataset under the current and future periods, with a resolution of 30″ (approximately 1 km^2^ on the ground) [58,59]. The future climate data for the 2050s (average for 2041–2060) and 2070s (average for 2061–2080) were downloaded from the Beijing Climate Center Climate System Modeling version 1.1 (BCC–CSM 1.1) for future model building [58,60,61]. In addition, we used four IPCC-CMIP5 representative concentration pathways (RCPs): one low mitigation gas emission scenario (RCP2.6), two moderate gas emission scenarios (RCP4.5 and RCP6.0), and one high greenhouse gas emission scenario (RCP8.5), which were labeled after the possible pathways of radiative forcing values (measured as +2.6, +4.5, +6.0, and +8.5 W/m^2^, respectively) in 2100 [62]. The vegetation type variable and land-use type were downloaded from the Resource and Environment Science and Data Center [63,64]. We downloaded the topographic variable (altitude) from the WorldClim dataset [58]. The water distance variables and human interference variables (distance to road and villages) were acquired from the 1:1 million National Catalogue Service for Geographic Information dataset [65]. Because land-use and distance to water are influenced by multiple socio-economic drivers, it is not easy to simply estimate the future condition of these two factors. Hence, we used the current datasets for land-use and distance to water as conservative prognoses for the future. Including dynamic and static variables can limit any extra uncertainty and improve the performance of the model under future climate scenarios [66,67].

In this study, all of these environmental variables were transformed into American Standard Code for Information Interchange (ASII) file format using ArcGIS 10.2 Conversion Tools. They were resampled at a 30″ spatial resolution. We used SPSS software 19 to reduce multicollinearity among these environmental variables by establishing the correlation coefficient matrix and eliminating highly correlated variables (r > 0.8) [68,69]. Ultimately, 13 environmental variables were selected (Table 1).

### 2.3. MaxEnt Model Prediction

The 87 occurrence site data and filtered environmental variable data were imported into MaxEnt software 3.3.3 [27]. In our model, we randomly selected 25% of the occurrence data for model testing, and the remaining 75% of the data were used for model training [27]. In addition, the number of iterations was set to 500 and 10,000 background points to reduce uncertainty. Other values were kept as default. The average value of 15 repeated modelings was selected as the final prediction result. The jackknife approach was used to analyze the contribution of each environmental variable to the model based on how significantly each environmental variable occupied the species distribution [70]. Meanwhile, we used the receiver operating characteristic (ROC) curve and the area under the ROC curve (AUC) to verify the accuracy of the model. The AUC value is not affected by the threshold, and the evaluation results are relatively objective. Therefore, it is widely used for the accuracy evaluation of SDMs [71]. The accuracy of model prediction is directly proportional to the AUC value, which ranges from 0 to 1. An AUC value of 0.50–0.60 indicates failure, 0.60–0.70 represents poor performance, 0.70–0.80 indicates moderate performance, 0.80–0.90 means the modeling results can be adopted, and a value >0.9 indicates high performance [27].

The results of the modeled species distribution are continuous values that vary between 0 and 1. We imported the predicted result of the model into ArcGIS10.2. We reclassified it to obtain four potential habitat categories: highly suitable habitat (*p* ≥ 0.6), moderately suitable habitat (0.4 ≤ *p* < 0.6), poorly suitable habitat (0.2 ≤ *p* < 0.4), and unsuitable habitat (*p* < 0.2). The area of each partition was also calculated.

### 2.4. Habitat Distribution Change and the Core Distributional Shifts

After using the current climate data to model the spatial extent of suitable habitat for the Eurasian Spoonbill, we obtained the future distribution of the species by performing model projections with eight future scenarios (RCP2.6-2050, RCP2.6-2070, RCP4.5-2050, RCP4.5-2070, RCP6.0-2050, RCP6.0-2070, RCP8.0-2050, and RCP8.5-2070). The change of suitable distribution area was obtained by comparing the distribution areas in the present and future periods through ArcGIS10.2. The results were classified into four categories: habitat loss, consistently unsuitable habitat, unchanged suitable habitat, and becoming suitable habitat. Afterward, we calculated the centroid of the suitable habitat in the current and future scenarios using the SDM toolbox, a Python-based GIS software10.7, to evaluate the trend of change in the suitable area [72].

## 3. Results

### 3.1. Model Performance and Potential Distribution of Current Wintering Habitat

**Figure 2:**
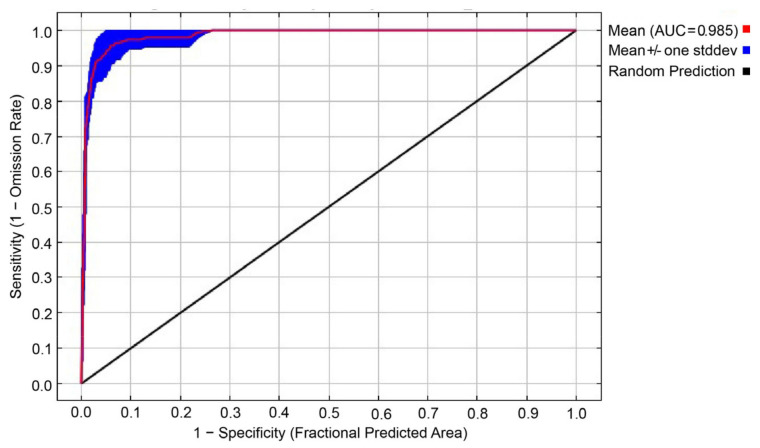
ROC curve and AUC value (note: ROC represents receiver operating characteristic; AUC represents the area under the ROC curve).

The AUC value of the model showed that the MaxEnt model performed well (AUC = 0.985) (Figure 2). This finding indicates that the MaxEnt model exhibited a good performance that can be used to predict the potential wintering habitat for the Eurasian Spoonbill. The current suitable habitat total area was 106,621 km^2^. The highly suitable area, moderately suitable area, and poorly suitable area were 22,197, 30,185, and 54,239 km^2^, representing 21%, 28%, and 51% of the suitable habitat area, respectively. The suitable habitat of white spoonbills is wetlands, including natural wetlands such as offshore and coastal areas, rivers, lakes, and marshes, as well as artificial wetlands such as reservoirs, rice paddies, salt farms, and fish farms.

**Figure 3:**
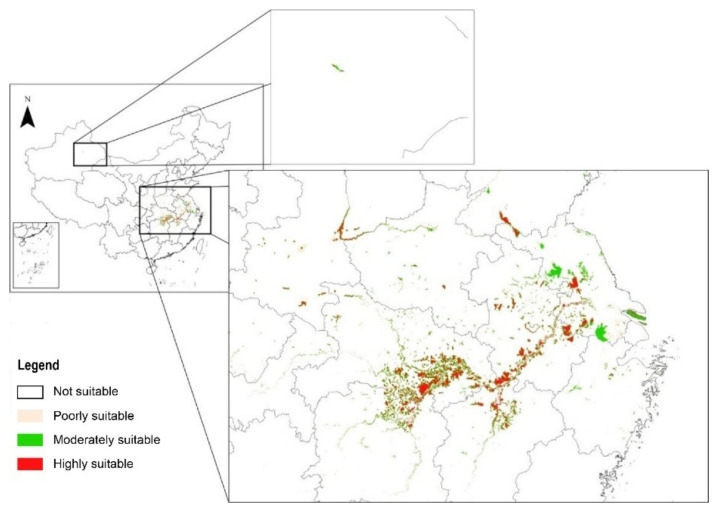
The current potential distribution of suitable areas for the Eurasian Spoonbill.

The suitable habitats are distributed in 21 provinces (autonomous regions and municipalities) in China, mainly located in Hubei, Hunan, Jiangxi, and Anhui provinces; a few are scattered in Jiangsu, Henan, Shaanxi, Shandong, and Xinjiang provinces; very few are scattered in Yunnan, Guangdong, Zhejiang, Shanghai, Chongqing, and Sichuan provinces (Figure 3).

### 3.2. Major Environmental Factors Affecting the Distribution of Wintering Spoonbills

The MaxEnt model’s internal jackknife test of factor importance showed that water_distance (52.2% contribution rate), precipitation of the driest quarter (Bio17, 15.3% contribution rate), altitude (Alt, 10.0% contribution rate), and mean temperature of the driest quarter (Bio9, 8.0% contribution rate) made the greatest contribution to the distribution model for the Eurasian Spoonbill. The cumulative contributions of these factors reached values as high as 85.5% (Table 2).

**Figure 4:**
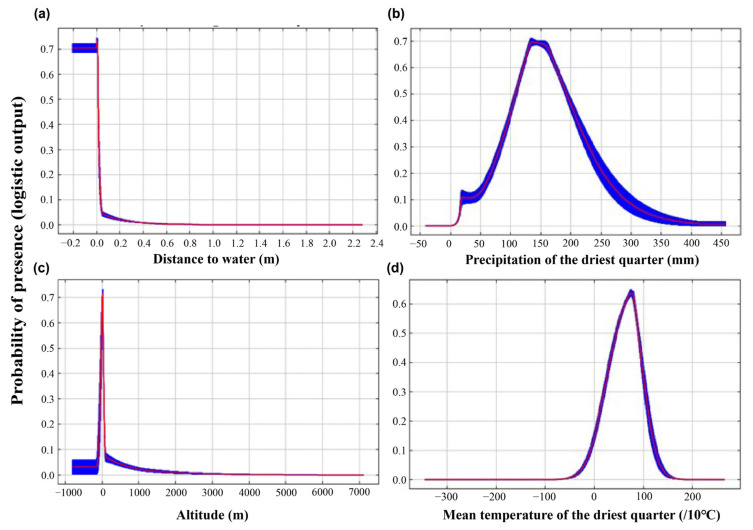
Response curves of probability of presence (based on the logistic output of the MaxEnt model) for the Eurasian Spoonbill (vertical axis) to Distance to water (**a**), Precipitation of the driest quarter (**b**), Altitude (**c**), and Mean temperature of the driest quarter (**d**); the curves are shown as means (red lines) with standard deviation (SD, blue buffers).

Figure 4 showed that the suitable altitude ranged from −100 m to 100 m. The highest suitability occurred when the area was near water. For hydrothermal conditions, the suitable range and optimal value of the precipitation of the driest quarter (Bio17) were from 110 mm to 190 mm. The suitable range of the mean temperature of the driest quarter (Bio9) was from 4 °C to 10 °C.

### 3.3. Future Changes in Suitable Habitat Area

**Figure 5:**
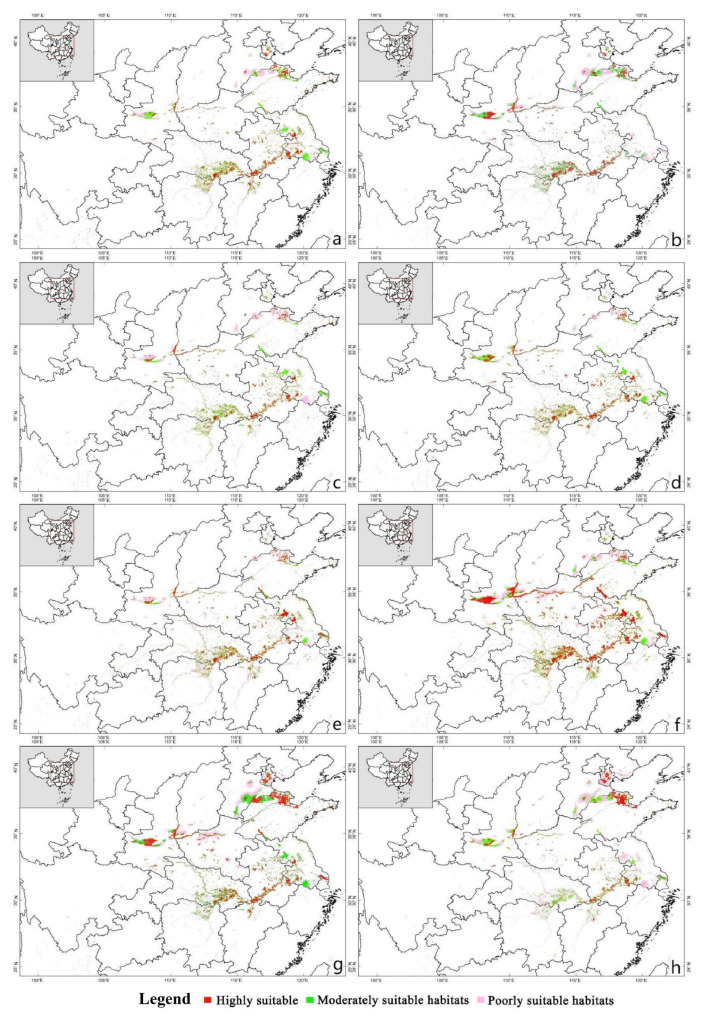
Potential wintering habitat of Eurasian Spoonbill under different climate change scenarios. (**a**) Climate scenario RCP2.6 in 2050; (**b**) Climate scenario RCP2.6 in 2070; (**c**) Climate scenario RCP4.5 in 2050; (**d**) Climate scenario RCP4.5 in 2070; (**e**) Climate scenario RCP6.0 in 2050; (**f**) Climate scenario RCP6.0 in 2070; (**g**) Climate scenario RCP8.5 in 2050; (**h**) Climate scenario RCP8.5 in 2070.

**Figure 6:**
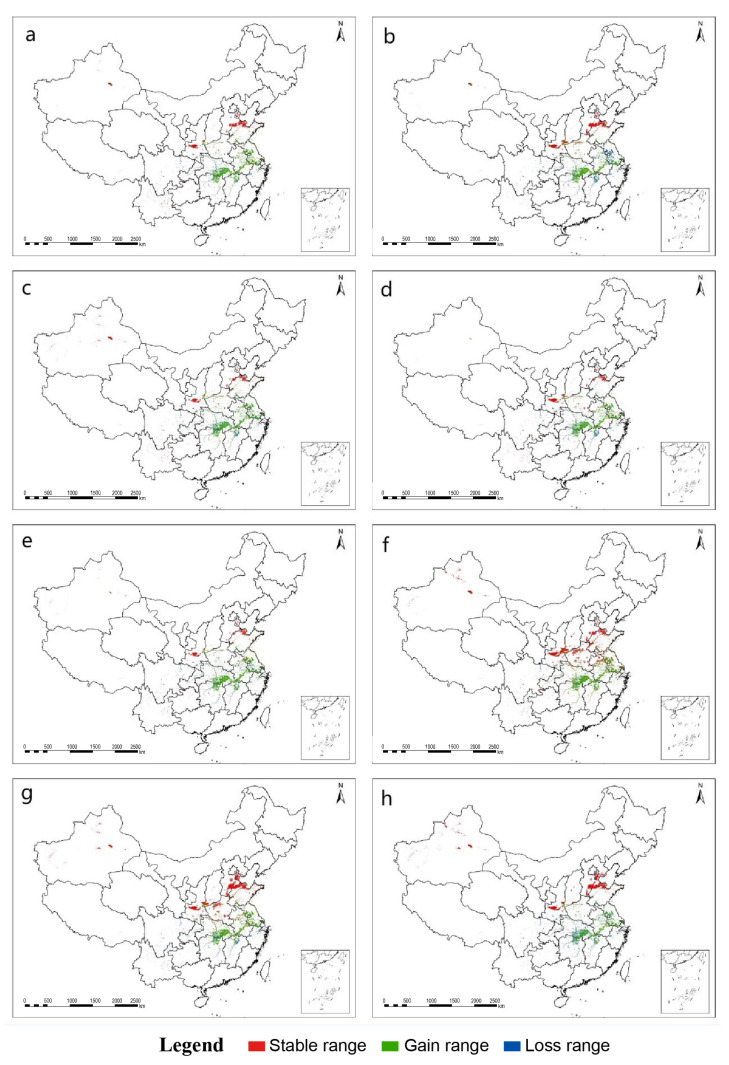
Spatial shifts for the Eurasian Spoonbill under different climate change scenarios. (**a**) Climate scenario RCP2.6 in 2050; (**b**) Climate scenario RCP2.6 in 2070; (**c**) Climate scenario RCP4.5 in 2050; (**d**) Climate scenario RCP4.5 in 2070; (**e**) Climate scenario RCP6.0 in 2050; (**f**) Climate scenario RCP6.0 in 2070; (**g**) Climate scenario RCP8.5 in 2050; (**h**) Climate scenario RCP8.5 in 2070.

The MaxEnt model predicted a dramatic range shift in the distribution of the suitable wintering habitat for Eurasian Spoonbill under four climate scenarios. The suitable distribution would expand to the northwest, while the suitable wintering habitat in the south would decrease. Compared with the current distribution, the suitable areas of northeastern Hunan, southeastern Hubei, Jiangxi, Jiangsu, and Anhui provinces would be lost. In contrast, the suitable areas of Shandong, Tianjin, Shaanxi, and the border with Henan Shanxi provinces greatly increase (Figure 5 and Figure 6).

The suitability of the Eurasian Spoonbill distribution range would improve with climate change (Table 3). Especially in the RCP6.0-2070s, the suitable wintering habitat was expected to increase by 77.28% (Table 4). In general, the suitable area showed a trend of increasing area and expanding northeast in the future.

### 3.4. The Shift of the Core Suitable Habitat Center under Different Climate Scenarios

**Figure 7:**
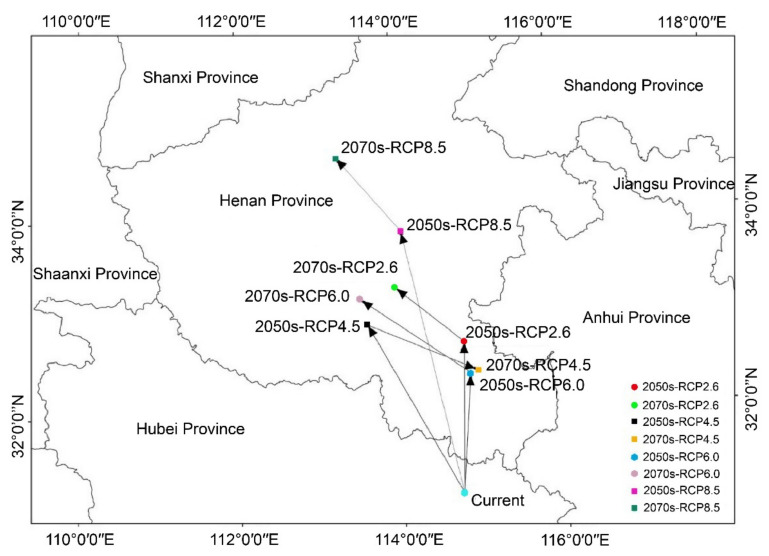
The centroid distributional shifts under different climate scenarios for the Eurasian Spoonbill.

The current habitat centroid of the Eurasian Spoonbill is at 114°43′ E and 31°12′ N in Hubei Province. The centroid of suitable habitat shifts to 114°48′ E and 32°43′ N in southeast Henan under RCP2.6-2050s. Moreover, it shifts to 113°59′ E and 33°17′ N in south central Henan under RCP2.6-2070s. Under RCP4.5-2050s, the centroid of suitable habitat is at 113°36′ E and 32°56′ N in southwest Henan. However, the centroid of suitable habitat shifts to the southeast (114°58′ E, 32°26′ N) under RCP4.5-2070s. The Eurasian Spoonbill migrates from southeast Henan (114°52′ E, 32°24′ N) under RCP6.0-2050s to west central Henan (113°32′ E, 33°14′ N) in the 2070s. The centroid of suitable habitat is at 114°6′ E, 33°53′ N in central Henan under RCP8.5-2050s. Finally, it shifts to northwest Henan (113°16′, E 34°39′ N) in the 2070s. Overall, the distribution center of the suitable habitat of the Eurasian Spoonbill generally moves from northeast Hubei to Henan from the present to the future. However, there was no significant correlation of the centroid shift observed between the different concentrations of greenhouse gas emissions (Figure 7).

## 4. Discussion

Our study provides the first look at the current and future species potential wintering distribution of the Eurasian Spoonbill in China. It shows that the suitable range changes dramatically under future climate scenarios, where the suitable habitat area would increase and shift to the northwest as a whole.

In this study, we found that the distance to water represents the environmental variable that contributes the most to the suitable wintering habitat and spatial distribution of the Eurasian Spoonbill. However, its effect on the probability of occurrence of the species is nearly constant. Only places close to water are suitable for the Eurasian Spoonbill. This finding is not very surprising because the Eurasian Spoonbill is a waterbird living and feeding in wetlands during winter [49,73]. The Eurasian Spoonbill is a specialist waterbird in shallow wetlands because it can only feed in water not deeper than 40 cm [74]. Furthermore, our results indicated that the suitable range of the precipitation of the driest quarter is from 80 mm to 230 mm. Thus, the precipitation of the driest quarter may limit the habitat selection of the Eurasian Spoonbill. Moreover, the mean temperature of the driest quarter may determine whether the temperature is suitable for the wintering Eurasian Spoonbill. For example, Poyang Lake and Dongting Lake are the main wintering areas for the Eurasian Spoonbill. The average temperature in January of Poyang Lake is 4.5 °C [75]. The average January temperature of Dongting Lake is 4.1 °C to 4.5 °C [76]. This finding is consistent with our results. Furthermore, this species prefers lower altitudes; this finding is also consistent with the results from a previous study [77]. It lives in wetlands below 100 m above sea level, and any significant change in temperature and precipitation may affect the potential distributions of the species, as shown in other research for other avian geographical distributions [78].

Climate change is one of the main factors affecting the distribution of birds [79,80]. The WWF (World Wide Fund for Nature) reported that global climate change threatens the survival of most birds [80,81]. Biologists need to predict the effect of climate change on species distribution, which has important implications for species conservation [2]. In our study, under a full dispersal assumption, we found that compared with the current situation, the average suitable wintering habitat area showed an increasing trend in the north. However, current suitable habitats will be lost, and some areas will experience a reduction in Eurasian Spoonbill habitat by up to 37.77% by 2070 These results are consistent with other studies; for example, Wu et al. reported that during the period from 1951 to 2010, more than 50% of the 14 species of birds in China migrated to the north, whereas Li et al. found that more than 40% of 108 species of birds in China will migrate approximately 90 km to the north by 2080 [82,83].

Birds are influenced by changes in temperature and precipitation [80]. Studies have shown that the average annual precipitation in China will increase (0%–20%), particularly in northern China and northwest China [84], and wetlands will provide more food and habitat for waterbirds [30]. In addition, climate warming will change the distribution pattern of birds and expand their distribution range [85]. This is consistent with our research results. At the same time, some studies have indicated that suitable habitats for some waterbirds decrease as the climate warms [86,87,88]. In extreme cases, the temperature in China is predicted to rise in the range of 2.5–5 °C by the end of the 21st century [89]. This scenario is likely to be one of the main contributors to the current loss of suitable habitat. We quantified the changes in the suitable wintering habitat of the Eurasian Spoonbill under four climate scenarios. We found that the area increases in comparison to the current distribution in the 2050s and 2070s, except in RCP4.5-2070s. No certain regular change is determined in the habitat area, probably because the relationship between temperature and precipitation in different climate scenarios is complex and nonlinear; this finding is similar to the findings of other studies [30,90,91], and the intrinsic reasons deserve further study in the future.

Our results indicated that some areas suitable for the Eurasian Spoonbill wintering at present (Jiangxi, Hunan Zhejiang, and Jiangsu) may not be suitable in the future because of climatic factors, such as dryness or high temperature. At the same time, in the future there will be some new suitable areas in the north and northwest (Shaanxi, north Shandong, Tianjin, the junction of Shaanxi, Shanxi, and Henan). Hence, climate change is an important factor affecting the overwintering distribution of birds [92,93]. When protecting the Eurasian Spoonbill and other waterbirds, we should pay attention to these areas and the phenomenon.

A large proportion of the population of the wintering Eurasian Spoonbill is mainly concentrated in the middle and lower reaches of the Yangtze River [94]. However, only some habitats are located in the protected area. These areas are under great pressure from human activities and habitat destruction. For instance, the wetland landscape pattern and ecological functions of Poyang Lake and Dongting Lake changed because of the expansion of water conservancy projects, agriculture, and aquaculture [95,96]. These changes may cause the Eurasian Spoonbill to move to smaller areas. Establishing protected areas is one of the effective means of protecting biodiversity and habitats. Therefore, some efforts, such as protecting the existing protected areas and establishing new reserves, should be undertaken in the areas identified as suitable habitats for Eurasian Spoonbills. At the same time, long-term surveys and monitoring in these areas can be considered in order to thoroughly understand the population status.

Climate change is an important factor, and bird species have responded to climate change [97]. It has also been shown that if the impact of climate change is not considered when establishing protected areas, some species may move out in the future [98]. Moreover, conservationists believe that it is very meaningful to consider the impact of short-term or long-term extreme weather when protecting and managing species in the context of climate change [99]. Therefore, we suggest integrating the current situation and future climate change effects when designing nature reserves and making management plans for the Eurasian Spoonbill or other waterbirds throughout China.

Many factors, such as interspecific relationships (competition and predation), land-use change, and other limited factors may affect the distribution of species. It is hard to model all impact factors. Although the MaxEnt model is widely used, the prediction results of this single model still have errors. Hence, in future research, we suggest that more factors affecting the distribution of species should be considered comprehensively, and the best model should be selected through comparative analysis of multiple models.

## 5. Conclusions

In this study, we predicted the distribution pattern and dynamic changes of the wintering Eurasian Spoonbill in response to climate change by using the MaxEnt model. Our results indicated that water distance, precipitation of the driest quarter, altitude, and mean temperature of the driest quarter were important factors shaping the distribution of the Eurasian Spoonbill. At present, suitable wintering habitats are mainly distributed in the middle and lower reaches of the Yangtze River in China. With climate change, the suitable wintering area showed an increasing trend in the future, and its suitable distribution would shift to the northwest as a whole. This suggests that species distribution could be affected by climate change. We suggest that the current situation and future impacts of climate change should be taken into account in nature reserve design and management planning for the Eurasian Spoonbill and other endangered waterbirds throughout China.

## Figures and Tables

**Table 1:** Environmental variables used to drive the MaxEnt model in this study.

Variables	Description	Unit
Bio2	Monthly mean diurnal temperature range	°C × 10
Bio3	Isothermality	Dimensionless
Bio6	Min. temperature of the coldest month	°C × 10
Bio9	Mean temperature of the driest quarter	°C × 10
Bio10	Mean temperature of the warmest quarter	°C × 10
Bio15	CV of precipitation	Dimensionless
Bio17	Precipitation of the driest quarter	mm
Alt	Altitude	m
Veg	Vegetation type	Dimensionless
LUCC	Land-use type	Dimensionless
Water_distance	Distance to water	m
Road_distance	Distance to road	m
Village_distance	Distance to village	m

**Table 2:** Relative contributions of the environmental variables to the MaxEnt model.

Variable	Contribution Rate (%)	Variable	Contribution Rate (%)
Water_distance	52.2	CV of precipitation	1.8
Precipitation of the driest quarter	15.3	Land-use	0.7
Altitude	10	Vegetation type	0.5
Mean temperature of the driest quarter	8	Village_distance	0.3
Min. temperature of the coldest month	6.8	Road_distance	0.1
Mean temperature of the warmest quarter	2.5	Isothermality	0.1
Monthly mean diurnal temperature range	1.8		

**Table 3:** Suitable distribution areas for Eurasian Spoonbill under different climate scenarios (note: RCP, representative concentration pathway. RCP2.6 represents the minimum greenhouse gas emission scenario, RCP4.5 represents the medium greenhouse gas emission scenario, RCP6.0 represents the high greenhouse gas emission scenario, RCP8.5 represents the highest greenhouse gas emission scenario).

Period	Climate Scenario	Highly Suitable		Moderately Suitable		Poorly Suitable		Suitable Area
		Area km^2^	Percentage %	Area km^2^	Percentage %	Area km^2^	percentage %	Area km^2^
Current		22,197	21	30,185	28	54,239	51	106,621
2050s	RCP2.6	22,658	18	39,725	32	62,191	50	124,574
	RCP4.5	17,939	16	31,966	29	59,669	55	109,574
	RCP6.0	23,486	21	29,409	26	59,735	53	112,630
	RCP8.5	39,491	23	48,611	28	83,625	49	171,727
2070s	RCP2.6	22,121	19	32,063	27	63,053	54	117,237
	RCP4.5	19,037	18	32,616	31	53,157	51	104,810
	RCP6.0	45,243	24	48,433	26	95,499	50	189,175
	RCP8.5	20,556	17	31,597	25	72,137	58	124,290

**Table 4:** Dynamics of changes in suitable wintering habitat area for the Eurasian Spoonbill under different climate scenarios (note: RCP, representative concentration pathway. RCP2.6 represents the minimum greenhouse gas emission scenario, RCP4.5 represents the medium greenhouse gas emission scenario, RCP6.0 represents the high greenhouse gas emission scenario, RCP8.5 represents the highest greenhouse gas emission scenario).

Climate Scenario	Year	Area (km^2^)				Proportion of Area (%)			
		Gain	Loss	Stable	Total Change	Gain	Loss	Stable	Total Change
Representative concentration Pathway (RCP)2.6	2050	40,320	21,680	85,737	18,640	37.82	20.33	80.41	17.49
	2070	46,764	35,805	71,565	10,959	43.86	33.58	67.12	10.28
Representative concentration Pathway (RCP)4.5	2050	31,611	27,459	79,735	4152	29.65	25.75	74.78	3.9
	2070	30,041	30,759	76,379	−718	28.18	28.85	71.64	−0.67
Representative concentration Pathway (RCP)6.0	2050	30,090	23,801	83,424	6289	28.22	22.32	78.24	5.9
	2070	91,603	9200	98,679	82,403	85.91	8.63	92.55	77.28
Representative concentration Pathway (RCP)8.5	2050	85,274	19,821	87,741	65,453	79.98	18.60	82.30	61.38
	2070	58,911	40,269	66,821	18,642	55.25	37.77	62.67	17.48

## Data Availability

Not applicable.
